# Influence of intrauterine administration of *Lactobacillus buchneri* on reproductive performance and pro-inflammatory endometrial mRNA expression of cows with subclinical endometritis

**DOI:** 10.1038/s41598-018-22856-y

**Published:** 2018-04-03

**Authors:** S. Peter, M. A. Gärtner, G. Michel, M. Ibrahim, R. Klopfleisch, A. Lübke-Becker, M. Jung, R. Einspanier, C. Gabler

**Affiliations:** 1 Institute of Veterinary Biochemistry, Freie Universität Berlin, Oertzenweg 19b, 14163 Berlin, Germany; 2 Institute for the Reproduction of Farm Animals, Bernauer Allee 10, 16321 Bernau, Germany; 3 Institute of Veterinary Pathology, Freie Universität Berlin, Robert-von-Ostertag-Straße 15, 14163 Berlin, Germany; 4 Institute of Microbiology and Epizootics, Freie Universität Berlin, Robert-von-Ostertag-Straße 7-13, 14163 Berlin, Germany

## Abstract

Potential beneficial effects of lactic acid bacteria on the genital health of cows become of particular interest when considering the importance of an optimal uterine health status for the success of breeding in dairy farming. Therefore, the aim of the present study was to analyse the influence of an intrauterine administration of the *Lactobacillus buchneri* DSM 32407 on reproductive performance, uterine health status, endometrial mRNA expression of pro-inflammatory factors of cows with signs of subclinical endometritis (**SCE**). *L. buchneri* DSM 32407 (n = 56; [**LAC**]) or a placebo (n = 60; [**PLA**]) was administered on day 24–30 postpartum. Endometrial cytobrush samples of cows with **SCE** were taken before the administration and at three following weeks (n = 16 cows each for **LAC**/**SCE** and **PLA**/**SCE**). A higher proportion of cows of the **LAC** and **LAC/SCE** group was pregnant after the first service and median days to conception for cows pregnant on day 200 pp were shorter. Three weeks after the administration, the endometrial mRNA expression of *CXCL1/2*, *CXCL3*, *CXCR2*, *IL1B*, *IL8* and *PTPRC* was lower in the **LAC/SCE** group compared with the **PLA/SCE** group. These findings suggest that the presence of *L. buchneri* DSM 32407 contributes to a uterine environment that results in a better reproductive performance.

## Introduction

Bovine subfertility is one of the major reasons for enormous economic losses in the dairy industry^1^. Impaired reproductive performance such as prolonged intervals from calving to conception can be associated with uterine diseases of the postpartum period, e.g. subclinical endometritis (**SCE**)^2,3^.

Current treatments of **SCE** include antimicrobials and prostaglandin (PG) F_2α_^4,5^. However, unwanted consequences of the administration of antimicrobials are residues in milk and meat^6^ and the potential spread of bacterial resistances^7^. Furthermore, the efficacy of a hormonal therapy with PGF_2α_ remains controversial. Administration of a single dose or 2 treatments with PGF_2α_ at 35 and 49 (±3) days in milk (DIM) did not affect the prevalence of **SCE** and purulent vaginal discharge^8,9^. The reproductive performance was neither improved by antibiotic nor PGF_2α_ treatment^9,10^. Therefore, an alternative strategy for the effective treatment of **SCE** to improve the fertility rates without the unwanted implications of the use of antimicrobials would be of great benefit.

In human medicine, an alternative to the use of antimicrobials for the treatment of bacterial vaginosis in women is the use of lactobacilli^11^. Characteristics of the Gram-positive lactobacilli are the capacity to produce acetic and lactic acid, hydrogen peroxide and bacteriocins, which are rated to be beneficial for the suppression of pathogenic bacteria^12^.

From samples of the bovine uterus, several *Lactobacillus* spp. were cultivable^13–16^ and also detected by metagenomic pyrosequencing of the 16S rRNA gene^17,18^. It was shown that co-culturing of bovine endometrial epithelial cells with *L. buchneri* (now registered as *L. buchneri* DSM 32407) up to a multiplicity of infection (MOI) of 10 did not affect the viability of epithelial cells^16^. In addition, the mRNA expression or release of pro-inflammatory factors was not influenced for up to 6 h and 48 h, respectively. In contrast, the presence of *L. ruminis* and *L. amylovorus* provoked a pro-inflammatory response of the epithelial cells. An early study indicated that lactobacilli have an immunostimulatory effect on the endometrium^19^. In that study, after the intrauterine administration of two live *Lactobacillus* spp., an infiltration with mostly mononuclear cells into the endometrium was observed and a colonization of the endometrium by the selected lactobacilli strains for up to 12 days was noted. However, the impact of an intrauterine administration beyond the endometrial infiltration with immune cells remains unclear, especially referring to the uterine health status.

One possibility to draw conclusions on the uterine health status is the analysis of the mRNA expression of pro-inflammatory factors such as interleukins, chemokines, and enzymes of the PG synthesis. An elevation of the mRNA expression of such selected factors in case of **SCE** was found in several studies^20–24^. In the present study, the exact same time points of the puerperium were chosen for sampling as in a previous study where the levels of mRNA expression of such factors showed correlations with **SCE**^24^. Therefore, the mRNA expression analysis of pro-inflammatory factors seemed suitable to support the evaluation of endometrial health.

The objective of the present study was to clarify the influence of the intrauterine administration of *L. buchneri* DSM 32407 on the reproductive performance of clinically healthy cows (**SCE** or healthy). Therefore, cows were divided into two groups on days 24–30 postpartum (pp) and either the autochthonous *L. buchneri* DSM 32407 or a placebo was administered intrauterine. In addition, all cows with **SCE** were monitored for their uterine health status and the endometrial mRNA expression pattern of selected pro-inflammatory factors on a weekly basis during the following days 31–51 pp.

## Materials and Methods

### Preparation of *L. buchneri* solutions for intrauterine administration

The strain *L. buchneri* DSM 32407 was isolated from a uterus of a healthy cow and stored long-term at −80 °C in 15% (v/v) glycerol in MRS broth (according to DeMan, Rogosa and Sharpe; Sigma-Aldrich, Steinheim, Germany)^16^. This stock was used for enrichment by cultivation under aerobic conditions in MRS broth (Sigma-Aldrich) at 37 °C for 48 h until the suspension reached an optical density of one at the wavelength of 600 nm. After centrifugation for 10 min at 15000 × g and resuspension in MRS broth with 50% (v/v) glycerol, 200 µl aliquots were stored at −80 °C until further use.

36–48 h prior to the preparation of the solutions for intrauterine administration, aliquots were thawed at room temperature. To calculate the number of colony forming units (cfu)/ml of the aliquots, serial dilutions were cultivated on Rogosa SL agar (Sigma-Aldrich) under microaerophilic conditions (Anaerocult C, Merck, Darmstadt, Germany) at 37 °C. Thawed aliquots were stored at 4 °C until the preparation of the solutions for intrauterine administration.

It was observed that the number of cfu/ml was stable for up to 48 h in the thawed aliquots (stored at 4 °C) and for 8 h in the prepared solutions for intrauterine administration (stored at room temperature).

*L. buchneri* solutions for intrauterine administration consisted of 1.5–2 × 10^10^ cfu in 20 ml 0.9% (w/v) isotonic saline solution (B. Braun, Melsungen, Germany) drawn up into a 20 ml plastic syringe (Injekt, B. Braun). Bacteria were prepared at the day of administration (08:00 a.m.), transported to the farm at room temperature and administered latest on the same day after a maximum of 8 h.

### Examination and enrollment of cows in this study

Lactating Holstein cows included in this study were kept at the Lehr- und Versuchsanstalt für Tierzucht und Tierhaltung e. V. in Groß Kreutz (Brandenburg, Germany), housing around 200 cows in freestall facilities with slotted floors and cubicles in accordance with the guidelines of the National Animal Welfare Legislation. Animal experimental procedures were approved by the relevant authorities of the state Brandenburg, Germany (Landesamt für Umwelt, Gesundheit und Verbraucherschutz; V3-2347-2-2012 and V3-2347-19-2013).

On days 24–30 pp (**E**xam **1** [**E1**]), cows were examined by inspection of the vulva, vaginoscopy, transrectal palpation and ultrasonography (Tringa Linear, Esaote, Köln, Germany) of the uterus and ovaries to determine their uterine health status and stage of the oestrous cycle as reported previously^24^.

All cows with an initial status of a clinical healthy uterus (no (muco)purulent discharge detectable in the vagina)^25^ were included in this study (n = 116; 30 primiparous and 86 multiparous). Cows with signs of clinical endometritis (**CE**) [(muco)purulent uterine discharge detectable in the vagina] at **E1** were excluded from the study.

If a corpus luteum was present, cows were considered as being in the luteal phase (n = 95). The absence of a corpus luteum and also a Graafian follicle defined cows to be either prior to their first ovulation after parturition or 1–3 days after ovulation (n = 21). Cows showing signs of oestrus (presence of clear and cohesive mucus discharged from the vulva and/or the presence of a Graafian follicle) were excluded to prevent that the applied bacteria would be flushed out by the mucus.

### Intrauterine administration of *L. buchneri* or placebo and collection of luminal endometrial epithelial samples

After the examination at **E1**, samples from the endometrial epithelium were obtained with the cytobrush technique from the uterine body as reported previously^20,26^. Three cytobrush samples were collected. The first cytobrush was used for cytological analysis by rolling the cytobrush on a clean glass microscope slide directly after sampling at the farm. The second cytobrush was collected for the isolation of total RNA and was placed in a cryotube, which was immediately immersed into liquid nitrogen and stored at −80 °C until further use. The third collected cytobrush was used for bacteriological analysis and therefore was placed in an Amies medium containing tube (Heinz Herenz, Hamburg, Germany) and transported to the laboratory at room temperature.

After the sampling, cows were randomly divided into two groups. The prepared *L. buchneri* solutions were administered intrauterine to cows of the first group (n = 56; *Lactobacillus buchneri* group [**LAC**]). 20 ml of isotonic saline solution 0.9% (w/v) per cow as a placebo were administered intrauterine to the cows of the second group (n = 60; placebo group [**PLA**]). Briefly, the metallic catheter passing the cervix for cytobrush sampling from the uterine body was hold in place after the collection of endometrial samples. A sterilized 62 cm long polytetrafluoroethylene tube with an internal diameter of 2 mm (Rotilabo, Carl Roth) was inserted through the catheter for the aseptic administration of the prepared *L. buchneri* solutions or the placebo.

After the transportation to the laboratory, slides for cytological analysis were prepared and evaluated as reported previously^24^. If the content of polymorphonuclear neutrophils (PMN) in the cytological sample was < 5%, the uterine health status of the cows was defined as healthy (**H**; n = 83). If the content was ≥5%, cows were classified as having **SCE** (n = 33)^20,27^. Within the **LAC** group (n = 56), 40 cows were classified as **H** and 16 cows were diagnosed with signs of **SCE**. Within the **PLA** group (n = 60), 43 cows were classified as **H** and 17 cows were diagnosed with **SCE**. Reproduction performance data of all cows treated with either *L. buchneri* or placebo were recorded and considered for statistical analysis.

### Monitoring of uterine health status and collection of luminal endometrial epithelial samples after E1

Cows with **SCE**, both from the **LAC** and the **PLA** group, were further monitored for their uterine health status and sampled for mRNA expression analysis except for one cow from the **PLA** group because of technical reasons. This results in a distribution of 16 cows per group (**LAC**/**SCE** and **PLA**/**SCE**).

Each of these cows was examined and sampled three more times in the same manner as described above at **E1** in weekly intervals on days 31–37 pp (**E**xam **2** [**E2**]), on days 38–44 pp (**E**xam **3** [**E3**]), and on days 45–51 pp (**E**xam **4** [**E4**]).

### Microbiological analysis

At **E1**, **E2** and **E4**, a cytobrush sample was taken for microbiological analysis from 31 cows of the **LAC** group (**LAC/SCE** n = 16 and **LAC/H** n = 15) and from 17 cows of the **PLA** group (**PLA/SCE** n = 15 and **PLA/H** n = 2), respectively. Within the **PLA/SCE** group, the sample at **E2** could not be taken from one cow for technical reasons. The samples were analysed by aerobic and anaerobic cultivation after direct inoculation of the cytobrush with the suitable agar plates and also after the enrichment in a medium with subsequent cultivation on agar as described previously^24^.

### Biopsy sampling and histopathological examination

Non-pregnant cows (n = 8), which were not in the phase around oestrus and without any signs of clinical endometritis, were sampled for histopathological examination. Briefly, the vulva was cleansed with dry paper towel. The biopsy instrument (Institute for the Reproduction of Farm Animals, Bernau, Germany) was inserted into the uterine body through the cervix. Endometrial tissue samples were taken from the uterine body, followed by intrauterine administration of 1.5–2 × 10^10^ cfu of *L. buchneri* in 20 ml 0.9% (w/v) isotonic saline solution (**B**/**LAC**; n = 5) or placebo (20 ml 0.9% (w/v) isotonic saline solution; **B**/**PLA**; n = 3) in each cow, respectively. The application procedure was the same as described above. Biopsy samples from each cow were taken again one week later.

Pieces of endometrial tissue were immediately immersed into tubes containing Bouin’s solution for transportation. Samples were embedded in paraffin. Two sections of 2 µm thickness each 1000 µm apart were prepared, stained with hematoxylin and eosin, and examined for the presence of immune cells, integrity of the mucosa and proliferation by an observer blinded to the administration of *L. buchneri* or placebo.

### Breeding management

The voluntary waiting period was 60 days pp for most cows included in this study. However, 14 cows (**LAC** n = 9 and **PLA** n = 5) were inseminated earlier. Heat detection was done by observation once daily (10:00 a.m.) and by cow activity measured automatically with a respactor (X-ponder, Nedap, Groenlo, Netherlands) and a pedometer (Acto, Insentec, Marknesse, Netherlands). Cows were artificially inseminated within 24 h when heat was detected with deep frozen semen from bulls with similar fertility. Pregnancy diagnosis was performed by transrectal palpation and ultrasonography 30 days after insemination.

### Isolation of total RNA and reverse transcription

Total RNA from cytobrush samples harvested from cows with signs of **SCE** was isolated using the RNeasy Plus Mini Kit (Qiagen, Hilden, Germany), stored at −80 °C, and the integrity of the obtained total RNA was validated as previously reported^24^.

For the removal of genomic DNA, a DNase treatment was performed before reverse transcription^28^. Single strand cDNA was generated from 100 ng total RNA per sample with the addition of 200 U RevertAid Reverse Transcriptase and 2.5 µM random hexamer primers (both Thermo Scientific, Schwerte, Germany) in a total volume of 60 µl^29^. For the confirmation of the absence of any genomic DNA or contaminations, samples without reverse transcriptase were also prepared as negative controls. Generated cDNA was stored in aliquots at −20 °C until further analysis.

### Quantitative polymerase chain reaction (qPCR)

qPCR was performed to evaluate the mRNA expression of the candidate genes as reported previously^29^ following the minimum information for publication of quantitative real-time PCR experiments (MIQE) guidelines^30^. The genes of interest that were measured by this method are chemokine ligand 1/2 (*CXCL1/2*), *CXCL3*, *CXCL5*, chemokine receptor 2 (*CXCR2*), interleukin 1 alpha (*IL1A*), interleukin 1 beta (*IL1B*), *IL6*, *IL8*, *IL10*, interleukin 1 receptor antagonist (*IL1RN*), prostaglandin-endoperoxide synthase 2 (*PTGS2*), prostaglandin E_2_ synthase 1 (*PTGES*), *PTGES3*, prostaglandin D_2_ synthase (*PTGDS*), tumour necrosis factor (*TNF*), matrix metallopeptidase 1 (*MMP1*), and protein tyrosine phosphatase, receptor type C (*PTPRC*). Primer pairs were synthesized by Eurofins Genomics (Ebersberg, Germany) and details are given in Supplement Table 1. A gradient-PCR was performed to determine the optimal annealing temperature of unpublished primer pairs and obtained amplicons were subjected to commercial DNA sequencing (GATC Biotech, Konstanz, Germany) to confirm 100% homology to the published bovine sequences^29^.

Using the Rotor Gene 3000 (Corbett Research, Mortlake, Australia), amplification of 1 µl cDNA per sample was carried out in the presence of 0.4 µM of each primer (forward and reverse) and 5 µl 2 × SensiMix SYBR Low-ROX (Bioline, Luckenwalde, Germany) in a total reaction volume of 10 µl. Denaturation at 95 °C for 10 min was followed by a three-step amplification in 45 cycles: denaturation at 95 °C for 15 s, annealing for 20 s (temperatures depicted in Supplement Table 1), and extension at 72 °C for 30 s. Subsequently, a melting curve program (50–99 °C) with continuous fluorescence measurement confirmed specific amplification. For the generation of a standard curve, a dilution series with known concentrations of the purified amplicons was amplified simultaneously. In comparison with these standard curves, transcript amounts of specific mRNA were calculated using the Rotor Gene 6.1 software (Corbett Research).

### Statistical analysis

Reproductive performance was described by proportion of cows sold/culled, proportion of cows pregnant, days to first service, conception at first service, days to conception, and services per pregnancy. Survival curves for the proportion of cows pregnant on day 200 pp in relation to the number of days pp were generated using the Kaplan-Meier survival analysis. For the comparison of the Kaplan-Meier survival curves, three different statistical tests were used. The Breslow test tends to perform best towards the early time points and the Log Rank test to the late time points of the investigated period after calving. The Tarone-Ware test tends to perform best for the middle of this period.

The Fisher’s exact test was used to calculate the incidence of cultivable bacteria in the **LAC** group in relation to the **PLA** group at **E2** and **E4**.

The obtained mRNA expression values of the genes of interest was normalized using the geNorm tool^31^. Succinate dehydrogenase complex, subunit A (*SDHA*) and suppressor of zeste 12 homolog (*SUZ12*) were chosen as reference genes. Inter-run calibration was performed based on 10 inter-run calibrator samples using formula 15′^32^. Box plots were generated presenting the median values with 50% of all data within the box. Outliers (circles; values between 1.5- and 3.0-fold the interquartile range) and extreme values (asterisks; values beyond 3.0-fold the interquartile range) were included in statistical analysis.

Normal distribution was tested with the Shapiro-Wilk test. Neither the percentages of the PMN nor the normalized values of mRNA expression of the genes of interest or the data for reproductive performance were normally distributed. The Mann-Whitney U test was used to analyse values of the mRNA expression comparing samples from the **LAC**/**SCE** group with samples from the **PLA**/**SCE** group at **E2**, **E3** and **E4** and for the data of reproduction comparing the **LAC** group with the **PLA** group and the **LAC/SCE** group with the **PLA/SCE** group. Fold changes of the mRNA expression levels were calculated as the approximate ratio of the mean value of mRNA expression for the **LAC**/**SCE** group to the mean value of the **PLA**/**SCE** group.

All statistical evaluations and the generation of the box plots were performed using IBM SPSS Statistics 20.0 (SPSS, Chicago, USA) and the level of significance was set at *P* ≤ 0.05. A tendency to significance was considered when *P* was <0.10.

## Results

### Reproduction performance data

Several data of the descriptive reproductive performance considering all treated cows (**SCE** and **H**) are shown in Table 1. The proportion of cows sold/culled before pregnancy was 25% both in the **LAC** group and in the **PLA** group. Two cows of the **PLA** group were sold before breeding and 27 cows were culled due to problems as follows: the locomotor system (**LAC** group: 1 cow; **PLA** group: 4 cows), the udder (**LAC** group: 11 cows; **PLA** group: 7 cows), the fertility (**LAC** group: 2 cows), or a low milk yield (**PLA** group: 2 cows). The proportion of cows becoming pregnant was 75% both in the **LAC** and in the **PLA** group. The number of cows inseminated at least once was 96 in total, including 9 cows that were culled before conception (**LAC** group: 4; **PLA** group: 5).

**Table 1:** Descriptive reproductive performance outcomes of 116 cows as well as of 33 cows with signs of **SCE** after the intrauterine administration of 1.5–2 × 10^10^ cfu of *L. buchneri* DSM 32407 diluted in 20 ml isotonic saline solution 0.9% (**LAC** group) or 20 ml isotonic saline solution 0.9% as a placebo (**PLA** group) on days 24–30 pp.

Variable	LAC group	PLA group	*P*-value	LAC/SCE group	PLA/SCE group	*P*-value
Number of cows	56	60		16	17	
Number of cows inseminated	46	50		12	17	
Number of cows sold/culled before pregnancy	14 (25%)	15 (25%)		6 (37.5%)	2 (11.8%)	
Number of cows pregnant	42 (75%)	45 (75%)		10 (62.5%)	15 (88.2%)	
Median days to first service	67	81	0.157	63	65	0.107
Conception at first service	45.7%	30%		60%	13.3%	
Services per pregnancy	1.9	2.4	0.087	1.7	3.3	0.012
Number of cows pregnant on day 200 pp	39	36		10	10	
Median days to conception (of cows pregnant on day 200 pp)	103	133	0.035	74	164	0.001

The median days to first service were lower and the first service conception rate was higher in the **LAC** group compared with the values of the **PLA** group. The **LAC** group had less services per pregnancy and significant shorter median days to conception compared with the data in the **PLA** group.

Similar differences were observed when only considering cows with signs of **SCE** (Table 1). 8 cows of the **SCE** group were culled due to problems as follows: the locomotor system (**LAC/SCE** group: 1 cow), the udder (**LAC/SCE** group: 5 cows; **PLA/SCE** group: 3 cows). The days to first service were similar. However, a higher proportion of cows of the **LAC/SCE** group was pregnant after the first service and less services per pregnancy were required compared with the **PLA/SCE** group. The median days to conception for cows pregnant on day 200 pp were 90 days shorter in the **LAC/SCE** group (*P* = 0.001) compared with the **PLA/SCE** group.

**Figure 1:**
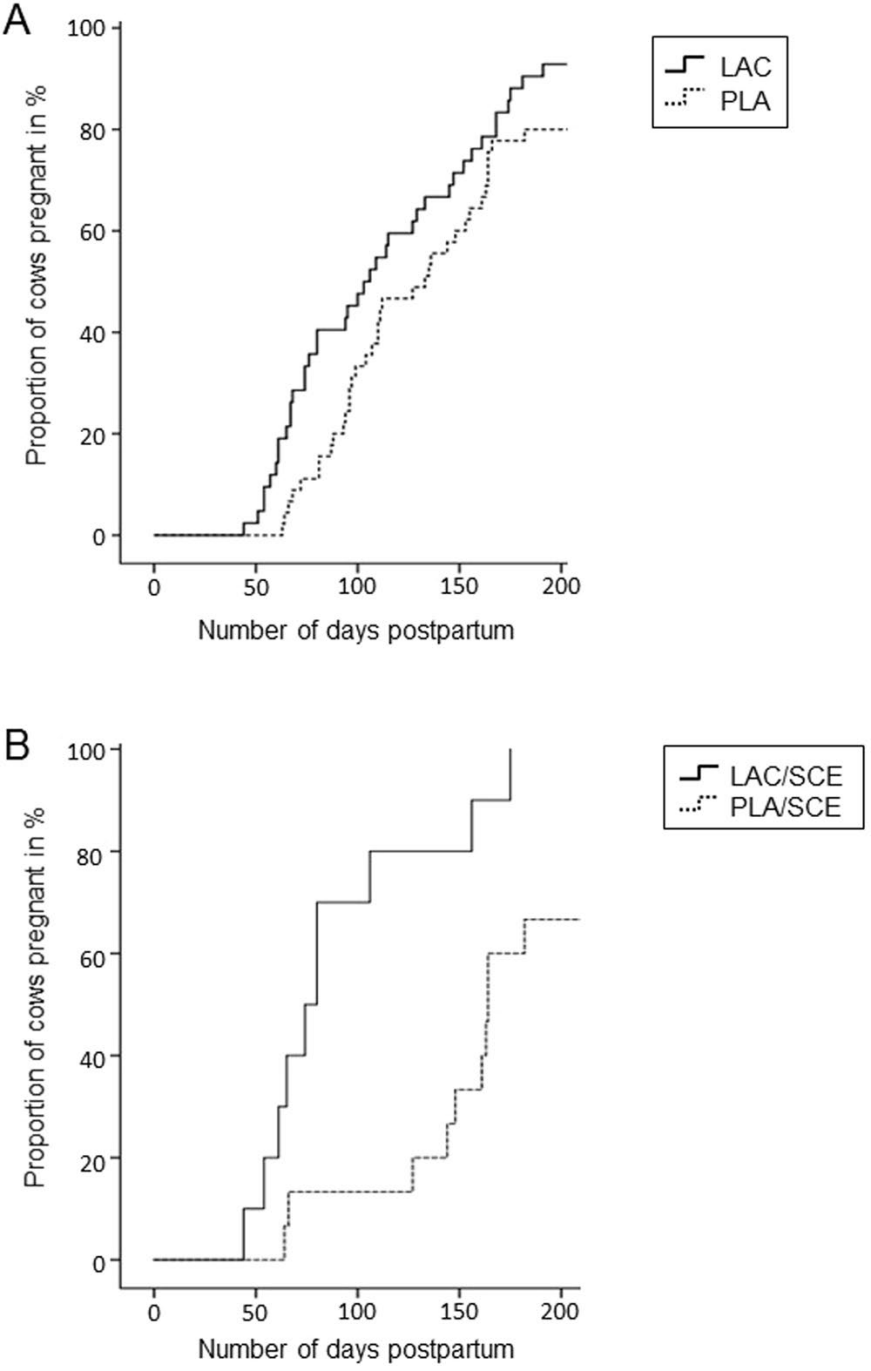
Kaplan-Meier survival curves for cows pregnant at day 200 pp. **(A)** The curves show the proportion of cows pregnant (in %) in relation to the number of days pp for the **LAC** group (n = 42; solid line) and the **PLA** group (n = 45; dotted line). The proportion of cows censored (not pregnant at day 200 pp) was 7.1% for the **LAC** group and 20.0% for the **PLA** group. *P* values: Breslow test (*P* = 0.035), Log Rank test (*P* = 0.06) and Tarone-Ware test (*P* = 0.047). **(B)** The curves show the proportion of cows pregnant (in %) in relation to the number of days pp for the **LAC/SCE** group (n = 16; solid line) and the **PLA/SCE** group (n = 17; dotted line). The proportion of cows censored (not pregnant at day 200 pp) was 0.0% for the **LAC/SCE** group and 33.3% for the **PLA/SCE** group. *P* values: Breslow test, Log Rank test and Tarone-Ware test (*P* = 0.001 each).

Kaplan-Meier survival analysis was performed to show survival curves for the proportion of cows pregnant on day 200 pp in relation to the number of days pp (Fig. 1A). Cows pregnant after day 200 pp were censored. The proportion of cows censored was 7.1% (3 out of 42) for the **LAC** group and 20% (9 out of 45) for the **PLA** group. Comparing these groups, cows from the **LAC** group became pregnant significantly earlier using the Breslow test (*P* = 0.035) and the Tarone-Ware test (*P* = 0.047), whereas the Log Rank test (*P* = 0.06) showed a tendency to significance. The Kaplan-Meier survival analysis is also presented for the cows with signs of **SCE** to show the proportion of cows pregnant on day 200 pp (Fig. 1B). The cows from the **LAC/SCE** group became significantly earlier pregnant compared with the **PLA/SCE** group using the Breslow test, the Tarone-Ware test and the Log Rank test (*P* = 0.001 each).

### Uterine health status for the LAC/SCE and PLA/SCE groups at E2, E3, and E4

The number of cows in the **LAC**/**SCE** group (n = 16 at **E1**) dependent on their current uterine health status (**H**/**SCE**/**CE**) was distributed at the later time points of monitoring as follows: **E2** (n = 10/5/1), **E3** (n = 12/2/2), and **E4** (n = 12/3/1). The distribution for the **PLA**/**SCE** group (n = 16 at **E1**) was as follows (**H**/**SCE**/**CE**): **E2** (n = 13/1/2), **E3** (n = 13/1/2), and **E4** (n = 11/2/3).

After day 31 pp, the uterine health status of most cows was diagnosed as **H** in both groups. However, there were still 5 cows with signs of **SCE** in the **LAC/SCE** group in comparison to only 1 cow in the **PLA/SCE** group at **E2**. At **E3**, all of these cows were diagnosed as **H**.

Two cows of the **PLA/SCE** group showed continuously signs of **CE** at **E2**, **E3**, and **E4**. In both groups, all other cows showing signs of **SCE** or **CE** at **E3** and/or **E4** developed these diseases after being healthy before, except for one cow of the **LAC/SCE** group that had signs of **CE** at **E2**, **SCE** at **E3**, and was healthy at **E4**.

### Content of PMN in endometrial cytobrush samples and microbiological analysis

**Figure 2:**
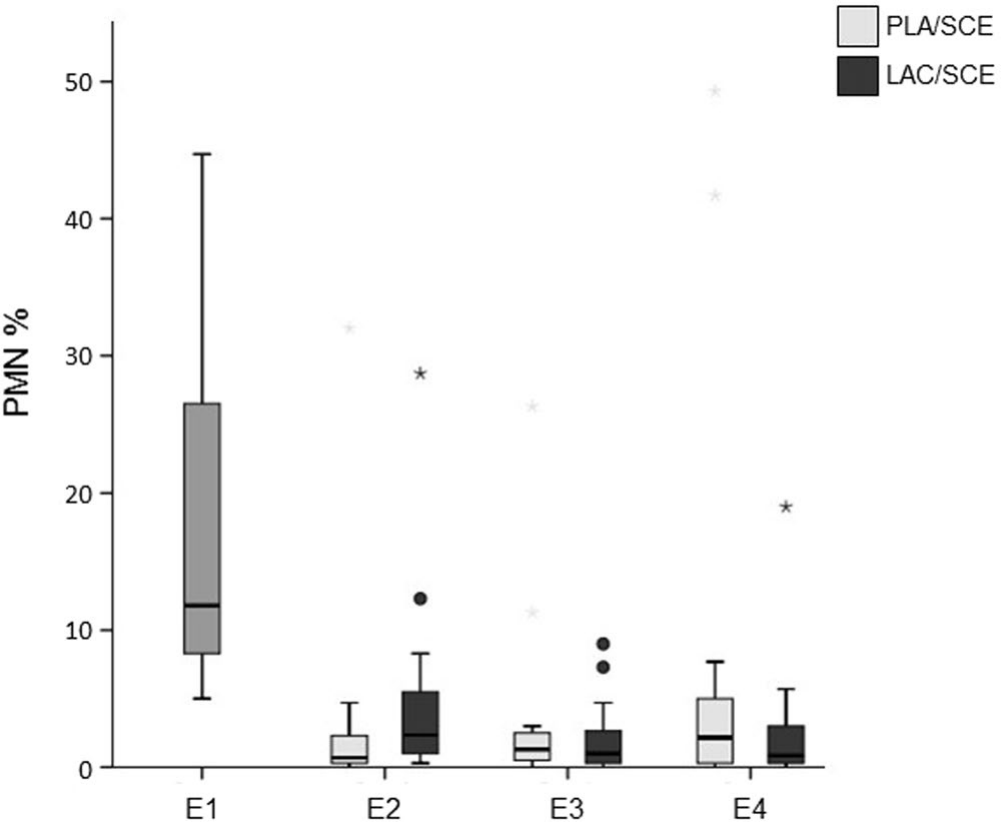
Percentages of PMN in bovine endometrial cytobrush samples harvested from dairy cows on days 24–30 pp (E1; n = 32), on days 31–37 pp (E2), on days 38–44 pp (E3), and on days 45–51 pp (E4). At E2, E3 and E4 cows were divided into the **PLA/SCE** (n = 16) and the **LAC/SCE** (n = 16) groups. Extreme values are diagrammed as asterisks, outliners as circles. Extreme values are not shown for one cow at E1 (73.3%) and for one cow of the **PLA/SCE** group at E2 (65%).

At **E1**, the content of PMN in the cytological preparations from endometrial cytobrush samples varied from 5–73.3%. At the later time points, the majority of samples had a percentage of PMN of <5%, both in the **PLA**/**SCE** and **LAC**/**SCE** groups (Fig. 2).

No significant differences were observed comparing the content of PMN of the **PLA**/**SCE** group with the **LAC**/**SCE** group at **E2**, **E3**, and **E4**. However, the number of PMN tended to be higher (*P* = 0.071) in the **LAC**/**SCE** group compared with the **PLA**/**SCE** group at **E2** (Fig. 2).

At **E1**, **E2** and **E4**, cultivable bacteria were *Trueperella pyogenes*, *Escherichia coli*, *Histophilus somni* and *Streptococcus uberis* from few samples of both the **PLA/SCE** and the **LAC** group. *Klebsiella pneumoniae* was only cultivable from one sample of **LAC/SCE** group. The number of samples that were positive for each bacterial species for these time points are presented in Supplement Table 2.

A significant difference was only observed for *H. somni* at **E2** (*P* = 0.032) with a higher number of positive samples in the **PLA/SCE** group compared with the **LAC** group.

### Evaluation of the mRNA expression analysis

Endometrial epithelial mRNA expression of all selected candidate genes was detected at all investigated time points in the postpartum period. However, mRNA expression for *IL1A*, *IL6, IL10, TNF*, *MMP1* and *PTPRC* could not be found in each sample.

At **E2**, **E3** and **E4**, most of the cows (**PLA**/**SCE** and **LAC**/**SCE**) were during their luteal phase, 1–3 days after ovulation or still prior to their first ovulation. However, seven cows were found during pro-oestrus at different time points (two at **E2**, two at **E3**, and three at **E4**) and two cows were found during oestrus at **E3**. The different oestrous cycle stages did not have a statistical significant influence on the mRNA expression values.

In the following text, mainly significant differences in normalized mRNA expression are described.

### mRNA expression of *CXCL1/2*, *CXCL3*, *CXCL5*, and *CXCR2*

**Figure 3:**
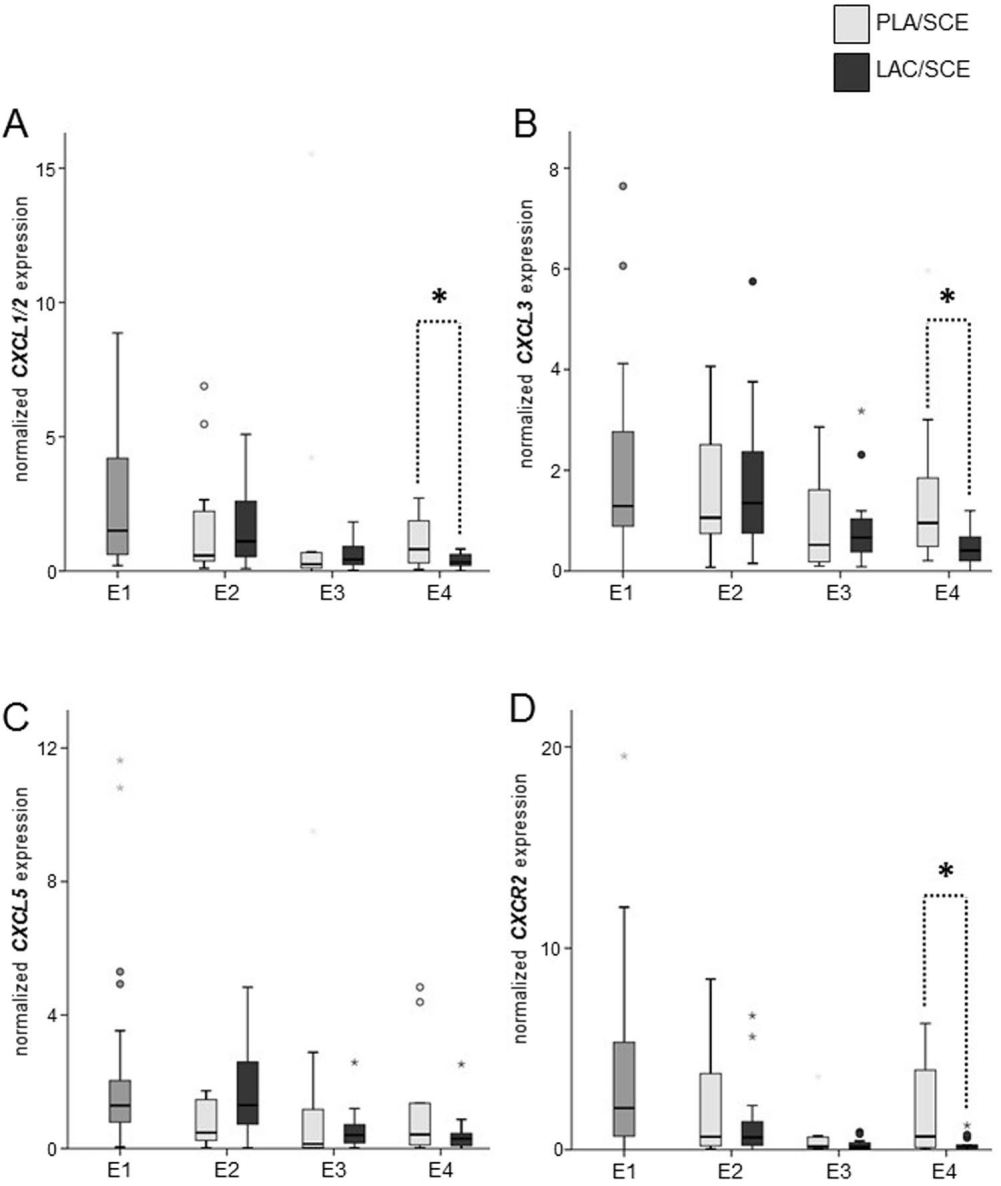
Normalized mRNA expression of (**A**) *CXCL1/2*, (**B**) *CXCL3*, (**C**) *CXCL5* and (**D**) *CXCR2* in bovine endometrial cytobrush samples harvested from dairy cows on days 24–30 pp (E1; n = 32), on days 31–37 pp (E2), on days 38–44 pp (E3), and on days 45–51 pp (E4). At E2, E3 and E4 cows were divided into the **PLA/SCE** (n = 16) and the **LAC/SCE** (n = 16) groups. Bold asterisks over dotted lines indicate significant differences between the groups (*P* ≤ 0.05). Extreme values are diagrammed as asterisks, outliners as circles. Extreme values are not shown for *CXCL1/2* obtained from three cows at E1 (17.69, 18.75, and 19.87) and from one cow of the **PLA/SCE** group at E4 (26.63); for *CXCL3* obtained from one cow at E1 (22.67); for *CXCL5* obtained from one cow of the **PLA/SCE** group at E2 (13.56) and E4 (21.72) and for *CXCR2* obtained from one cow of the **PLA/SCE** group at E2 (28.62), E3 (110.76), and E4 (109.09).

Primers named *CXCL1/2* are specific for *CXCL1* as well as for *CXCL2*^33^. At **E4**, *CXCL1/2* and *CXCL3* mRNA was seven- and threefold more highly expressed in the **PLA**/**SCE** group compared with the **LAC**/**SCE** group, respectively (Fig. 3A–B). In contrast, the mRNA expression pattern of *CXCL5* did not show significant differences between the **PLA**/**SCE** and **LAC**/**SCE** groups at all time points (Fig. 3C). However, a tendency (*P* = 0.07) was observed at **E2** when the *CXCL5* mRNA expression was higher in the **LAC**/**SCE** group compared with the **PLA**/**SCE** group.

Similar to *CXCL1/2* and *CXCL3*, the mRNA expression of *CXCR2* in luminal endometrial epithelium samples was affected at **E4**. *CXCR2* mRNA was 34-fold more highly expressed in the **PLA**/**SCE** group compared with the **LAC**/**SCE** group (Fig. 3D).

### mRNA expression of *IL1A*, *IL1B*, *IL6*, *IL8*, *IL10*, and *IL1RN*

**Figure 4:**
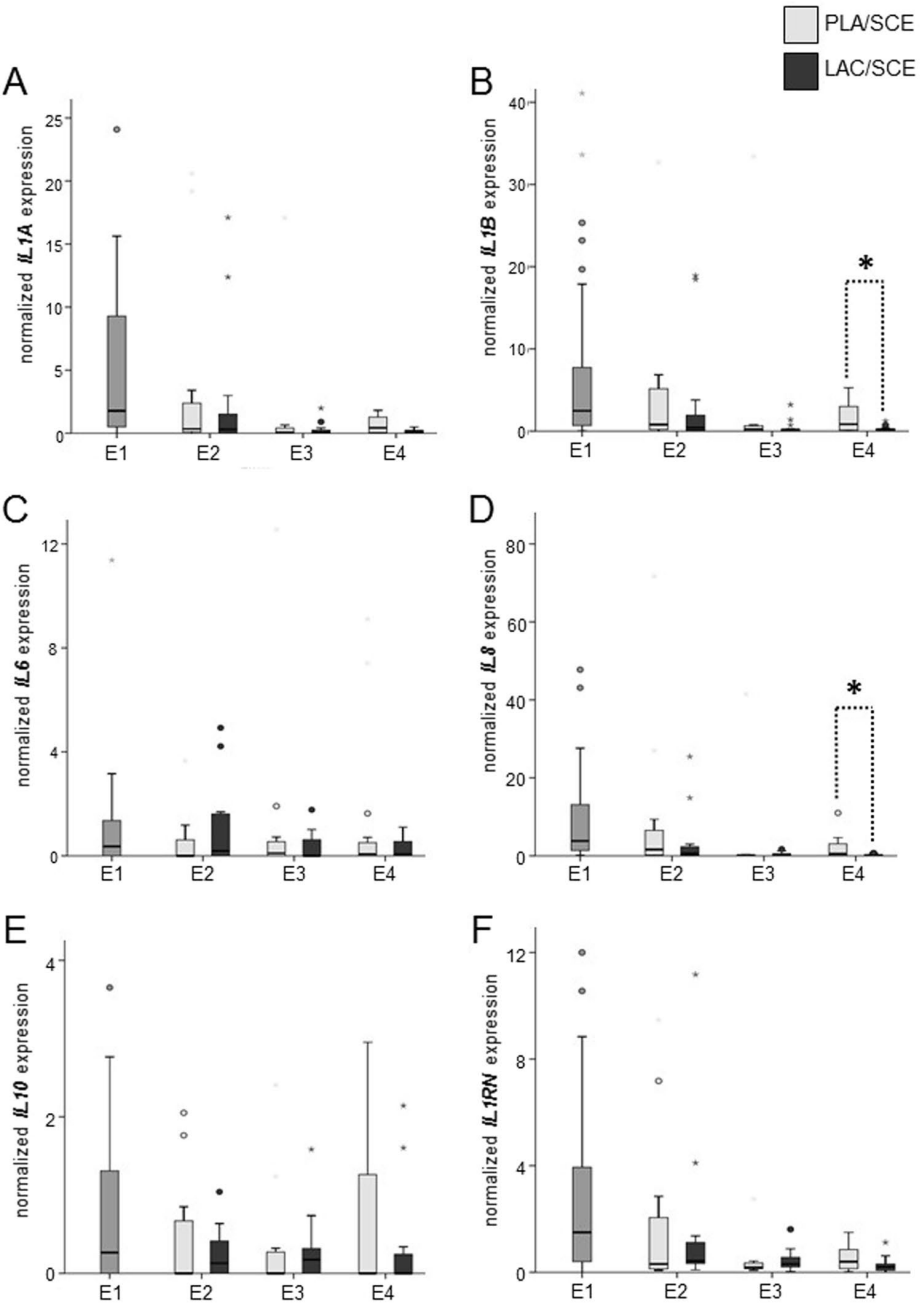
Normalized mRNA expression of (**A**) *IL1A*, (**B**) *IL1B*, (**C**) *IL6*, (**D**) *IL8*, (**E**) *IL10* and (**F**) *IL1RN* in bovine endometrial cytobrush samples harvested from dairy cows on days 24–30 pp (E1; n = 32), on days 31–37 pp (E2), on days 38–44 pp (E3), and on days 45–51 pp (E4). At E2, E3 and E4 cows were divided into the **PLA/SCE** (n = 16) and the **LAC/SCE** (n = 16) groups. Bold asterisks over dotted lines indicate significant differences between the groups (*P* ≤ 0.05). Extreme values are diagrammed as asterisks, outliners as circles. Extreme values are not shown for *IL1A* obtained from one cow at E1 (198.24) and from one cow of the **PLA/SCE** group at E3 (64.07) and E4 (94.13); for *IL1B* obtained from one cow of the **PLA/SCE** group at E2 (79.07), E3 (549.78), and E4 (223.11); for *IL6* obtained from two cows of the **LAC/SCE** group at E2 (116.41) and E3 (13.87); for *IL8* obtained from two cows at E1 (167.56 and 144.01) and from one cow of the **PLA/SCE** group at E3 (112.67) and E4 (143.67); for *IL10* obtained from one cow at E1 (14.2) and from one cow of the **PLA/SCE** group at E4 (18.7) and for *IL1RN* obtained from one cow at E1 (20.89) and from one cow of the **PLA/SCE** group at E3 (30.99) and E4 (19.59).

No significant differences were observed for the contents of *IL1A* mRNA in cytobrush samples of the **PLA**/**SCE** group compared with the samples of the **LAC**/**SCE** group during all time points (Fig. 4A). In addition, a decrease of the *IL1A* mRNA expression from E1 to the later time points was observed. In contrast, the mRNA expression of *IL1B* at **E4** was higher (*P* = 0.05) in samples from cows of the **PLA**/**SCE** group compared with the samples obtained from the **LAC**/**SCE** group (Fig. 4B).

*IL6* mRNA content was similar during the investigated period of the puerperium in all groups (Fig. 4C).

However, *IL8* mRNA was about 50-fold more highly expressed in endometrial samples from cows of the **PLA**/**SCE** group compared with the **LAC**/**SCE** group at **E4** (Fig. 4D).

*IL10* and *IL1RN* transcript amount did not differ significantly between the different treatment groups (Fig. 4E–F). However, *IL1RN* mRNA expression tended to be higher (*P* = 0.07) at **E4** in samples of the **PLA**/**SCE** group compared with samples of the **LAC**/**SCE** group.

### mRNA expression of *TNF, MMP1*, and *PTPRC*

**Figure 5:**
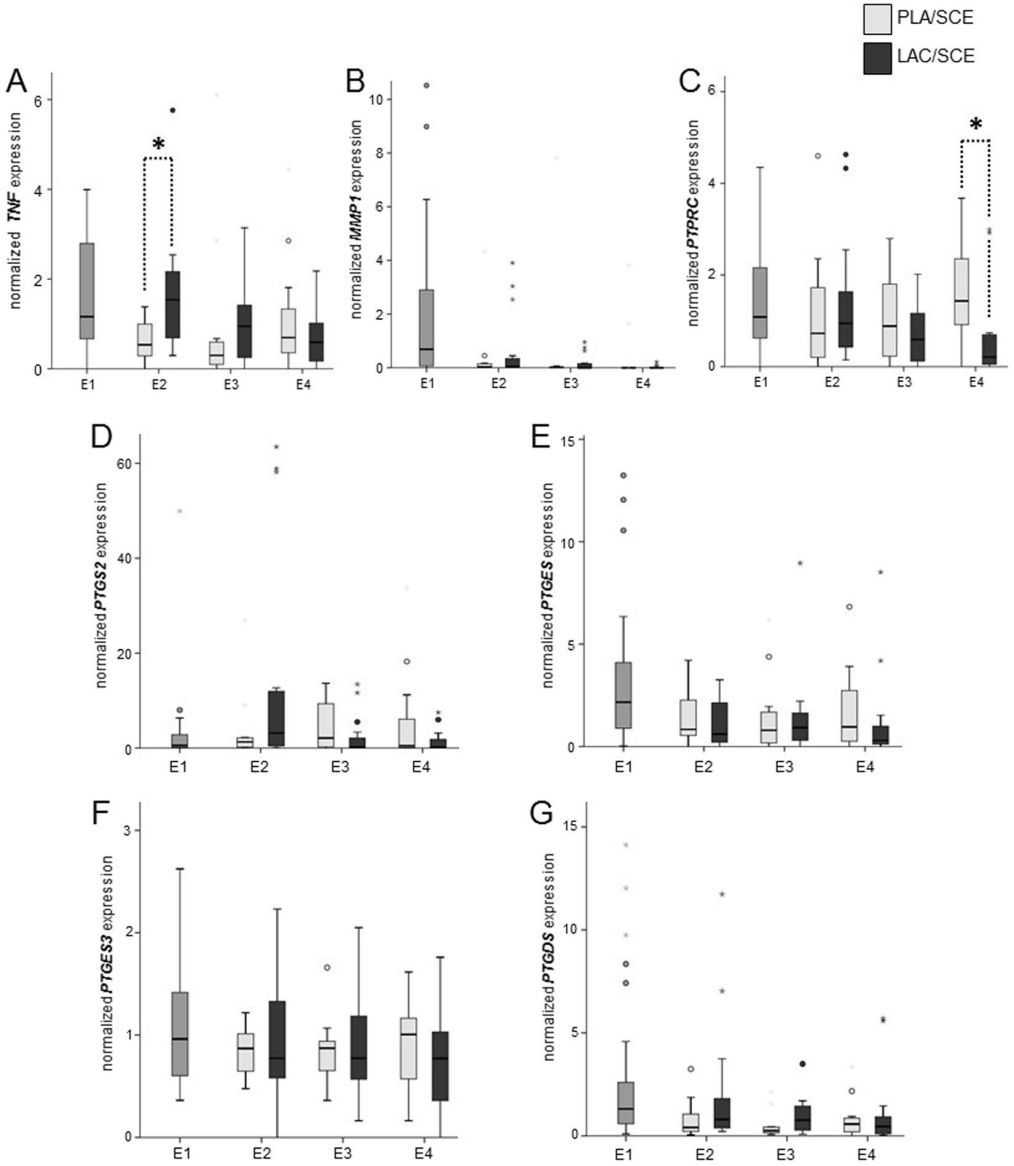
Normalized mRNA expression of (**A**) *TNF*, (**B**) *MMP1*, (**C**) *PTPRC*, (**D**) *PTGS2*, (**E**) *PTGES*, (**F**) *PTGES3* and (**G**) *PTGDS* in bovine endometrial cytobrush samples harvested from dairy cows on days 24–30 pp (E1; n = 32), on days 31–37 pp (E2), on days 38–44 pp (E3), and on days 45–51 pp (E4). At E2, E3 and E4 cows were divided into the **PLA/SCE** (n = 16) and the **LAC/SCE** (n = 16) groups. Bold asterisks over dotted lines indicate significant differences between the groups (*P* ≤ 0.05). Extreme values are diagrammed as asterisks, outliners as circles. Extreme values are not shown for *TNF* obtained from two cows at E1 (14.83 and 11.06) and from one cow of the **PLA/SCE** group at E4 (13.44); for *MMP1* obtained from one cow at E1 (56.61); for *PTPRC* obtained from one cow at E1 (23.53) and from one cow of the **PLA/SCE** group at E2 (12.86); for *PTGS2* obtained from one cow at E1 (138.26); for *PTGES3* obtained from one cow at E1 (6.16) and for *PTGDS* obtained from one cow of the **PLA/SCE** group at E4 (23.04).

The mRNA expression of *TNF* in endometrial epithelium cells was affected at **E2** by the treatment (Fig. 5A). *TNF* mRNA was threefold more highly expressed in samples obtained from cows of the **LAC**/**SCE** group compared with samples of the **PLA**/**SCE** group.

*MMP1* mRNA contents did not differ between the **PLA**/**SCE** group compared with the **LAC**/**SCE** group at all time points (Fig. 5B).

Furthermore, *PTPRC* mRNA was threefold more highly expressed in the endometrial samples of the **PLA**/**SCE** group compared with the samples of the **LAC**/**SCE** group at **E4** (Fig. 5C).

### mRNA expression of *PTGS2*, *PTGES*, *PTGES3*, and *PTGDS*

No significant differences of the mRNA expression were found in the transcript amounts in samples obtained from the **LAC**/**SCE** group compared with samples of the **PLA**/**SCE** group at **E2**, **E3** and **E4** for the analysed key enzymes of the PG synthesis as follows: *PTGS2*, *PTGES*, *PTGES3*, and *PTGDS* (Fig. 5D,G). However, the **LAC**/**SCE** group tended to have a higher *PTGDS* mRNA expression (*P* = 0.095) at **E3** compared with the **PLA/SCE** group (Fig. 5G).

### Histopathology of biopsy samples

**Figure 6:**
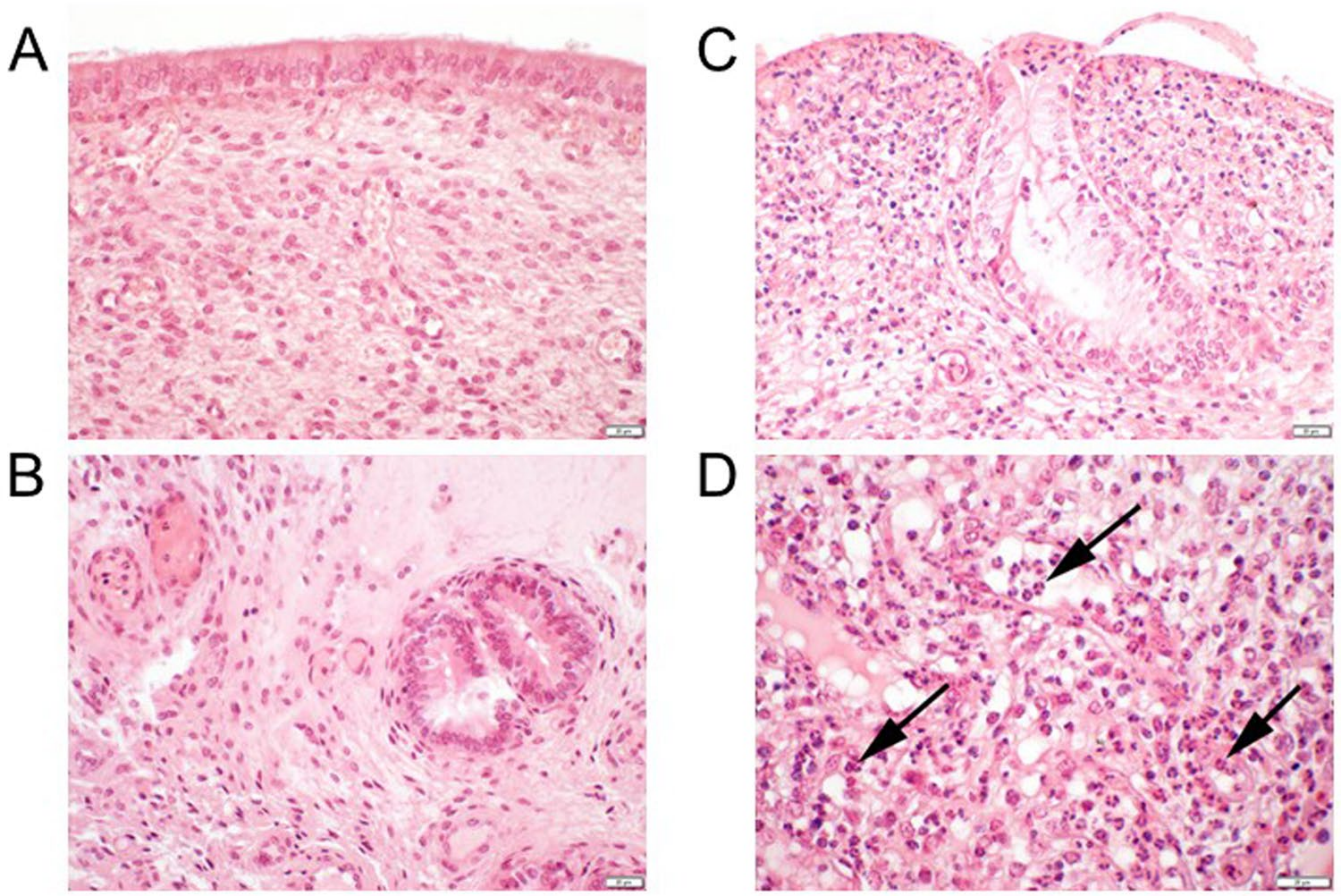
Biopsy samples of the endometrium of one cow from the **B**/**LAC** group. Hematoxylin and eosin staining; 2 µm thickness. (**A** and **B**) prior to the intrauterine administration of *L. buchneri* DSM 32407; no infiltration of immune cells, integrity of the mucosa and status of proliferation without pathological findings. (**C** and **D**) 7 days after the intrauterine administration of *L. buchneri *DSM 32407; moderate mainly granulocytic infiltration (arrows) and moderate multifocal degeneration.

No infiltration with immune cells was observed in all biopsy samples taken before the intrauterine administration of the *L. buchneri* solutions or the placebo, respectively. In addition, the integrity of the mucosa and the status of proliferation were without pathological findings (Fig. 6A,B).

One week after the intrauterine administration of the *L. buchneri* solutions or the placebo, respectively, all biopsy samples of the **B**/**PLA** group and one sample of the **B**/**LAC** group were judged identical to the samples that were taken previously to the administration. In the **B**/**LAC** group, a minimal granulocytic infiltration was observed in one sample, a minimal lymphocytic infiltration was observed in two samples, and a moderate mainly granulocytic infiltration was observed in one sample. This sample also showed a moderate multifocal degeneration (Fig. 6C,D). The integrity of the mucosa and the status of proliferation were without pathological findings in all other samples from the **B**/**LAC** group.

## Discussion

In a former study was shown that the strain *L. buchneri* DSM 32407 did neither influence the viability of endometrial epithelial cells nor provoke a pro-inflammatory response in contrast to other *Lactobacillus* strains^16^. Pathogenic (*Trueperella pyogenes*) or potential pathogenic strains (*Bacillus pumilus*) caused cell death even within 16–24 h^33,34^. Therefore, the strain *L. buchneri* DSM 32407 seems suitable to serve as a probiotic strain within the bovine uterus to modulate the immune response and the reproductive performance of dairy cows. The results of the present study support the hypothesis that lactobacilli improve the genital health resulting in better fertility rates, which are negatively influenced by inflammatory processes in the bovine uterus caused by pathogenic bacteria^35–37^.

A beneficial impact on reproductive performance, in particular on the interval from calving to conception, was observed after the intrauterine administration of *L. buchneri* DSM 32407. This might be associated to the down-regulated local immune system on days 45–51 pp, an indication for a healthy uterus at a time of the puerperium closer to first insemination^24^. Supporting this assumption of a positive impact on reproductive performance, one other study revealed that the presence of lactobacilli in the bovine uterus is related to a better pregnancy rate^17^. The percentage of cows positive for lactobacilli at 35 days in milk (DIM) was significantly higher in cows that were pregnant by 200 DIM compared with the group of cows that were not pregnant by that time. Interestingly, a similar effect was observed in a recent study that investigated the human endometrial microbiota. It showed that women with a *Lactobacillus*-dominated-microbiota (>90%) have higher chances of implantation, pregnancy and live birth after *in vitro* fertilization^38^. In this context, a positive effect of intrauterine lactobacilli on fertility might also be related to stimulatory effects on the blastocyst around the time of implantation. *In vitro* experiments showed that *L. acidophilus* culture supernatant positively influenced the growth and development of bovine embryos^39^.

Such influence on bovine genital health was additionally observed in recent studies. The weekly administration of a mixture of lactobacilli (LAB) into the vagina from two weeks before until four weeks after parturition (six treatments) decreased the occurrence of purulent vaginal discharge in dairy cows at week three pp^40^. In the same study, survival analysis revealed shorter calving to pregnancy intervals for the group of multiparous cows treated intravaginally with LAB. In two related studies, the same mixture of LAB were administered intravaginally only three or two times. Treated cows had a faster uterine involution and a lower incidence of uterine infections, cows with three treatments resumed ovarian cyclicity earlier and cows with two treatments had fewer days open^41,42^. However, a recent study showed that the number of intrauterine counted lactobacilli did not differ compared with controls when lactobacilli were intravaginal administered^43^. In contrast to the mentioned studies, the present study focused on the treatment of cows with signs of **SCE**, which have impaired reproductive performance and are difficult to detect^44^. In addition, the present study revealed that only one intrauterine application improved the reproductive performance tremendously. It was shown that cows, even healthy or with signs of **SCE**, showed a better reproductive performance after the treatment.

The improved fertility may be obtained because an intrauterine administration of *L. buchneri* DSM 32407 initially stimulates the local immune system. One week after the administration, at a time when the uterine health status of most cows recovered to health, cows of the **LAC**/**SCE** group tended to have higher percentages of PMN in endometrial samples. These findings are supported by an earlier study that observed the endometrial infiltration with immune cells for up to 12 days after the intrauterine administration of lactobacilli to clinically healthy cows and stated a possible stimulatory effect on endometrial defence mechanisms^19^.

The higher mRNA expression of *TNF* in the **LAC**/**SCE** group one week after the intrauterine administration of *L. buchneri* DSM 32407 supports these findings on a molecular basis. TNF is a pro-inflammatory cytokine that is produced by monocytes and/or macrophages infiltrating to the site of an inflammation^45^. It was found to be more highly expressed in the bovine endometrium during an inflammatory process, which reflects an activated immune system within the uterus^46^. The stimulation of cells with TNF results in the synthesis of CXCL5^47,48^, which explains that *CXCL5* mRNA contents tended to be higher in the **LAC**/**SCE** group at this time point. CXCL5 is a chemoattractant responsible for mediating neutrophil recruitment during inflammation and infection and binds to CXCR2, which is present especially on the surface of immune cells, e.g. PMN^49,50^.

In addition, the histopathology of endometrial biopsy samples of cows showed an infiltration with immune cells only in cows of the **B**/**LAC** group one week after the intrauterine administration. These biopsy data were supported by an *in vitro* study showing that *L. buchneri* DSM 32407 did not affect the viability of bovine endometrial epithelial cells in this short-time co-culturing experiments with a MOI up to 10^16^. However, this study only focused on the short-term influence of *L. buchneri* DSM 32407, whereas the results of the present study provide more information about the long-term effects *in vivo*.

Although there is an indication of an activated local immune system, the mRNA expression levels of the other investigated pro-inflammatory factors were not significantly influenced seven days after the intrauterine administration of *L. buchneri* DSM 32407. However, it is possible that the mRNA expression of these factors increased earlier than seven days after the administration, previous to the influx of immune cells.

In the early puerperium, an up-regulation of the local immune system for the clearance of invaded pathogenic bacteria is regarded as a physiological process^51^. In this context, it was observed that cows with a high PMN infiltration within the uterus during the first week pp have a better fertility compared with cows, which a have lower PMN infiltration^52^. Bacterial contaminations of the bovine uterus are almost inevitable with a prevalence of around 90%^53^ with pathogenic bacteria being the main cause for the development of endometritis^54,55^. The immunostimulatory effects for lactobacilli were also observed in earlier studies^19,56,57^ and the present study also suggests that lactobacilli are supportive for the containment of these pathogenic bacteria. It was also shown that a distinct lactobacilli strain alone or in combination with other reduced an *E. coli* infection and affected the pro-inflammatory reaction in bovine endometrial cells *in vitro*^58,59^.

In this context, there is indication that lactobacilli produce mitogenic and chemotactic factors. Culture supernatant of lactobacilli had strong pro-inflammatory properties, inducing the influx of PMN, the proliferation of macrophages and lymphocytes and the production of TNF by macrophages^60^. The competition with pathogens for limited nutrients also has to be taken into account^61^.

An early containment of pathogens might subsequently reduce local inflammatory processes in the endometrium and has beneficial effects on the genital health status. Although no different effects of the administration of *L. buchneri* DSM 32407 or the placebo on the clinical uterine health status of the **LAC**/**SCE** and **PLA**/**SCE** groups were observed, the present results of the mRNA expression analysis support this assumption. Three weeks after the intrauterine administration of *L. buchneri* DSM 32407 or the placebo, the mRNA of *CXCL1*/*2, CXCL3*, *CXCR2*, *IL1B*, *IL8* and *PTPRC* was lower expressed in the **LAC**/**SCE** group compared with the **PLA/SCE** group. These pro-inflammatory factors were higher expressed in cows with signs of **SCE** and/or **CE** and thereby related to uterine health problems in a recent study at the same time point^24^ and in previous studies at earlier time points of the puerperium^21,23,33^. The mRNA data of the present study thereby indicates that the endometrial immune system of cows treated with *L. buchneri* is downregulated three weeks after its administration to the uterus. Unfortunately, the present study design does not allow a satisfying explanation on why it takes three weeks to a noticeable effect on the immune system. In future studies, it would be of great benefit to investigate the endometrial microbiota by pyrosequencing before and after the administration of *L. buchneri* to better understand the time-dependent changes and possible correlations to the effects on the immune system.

In conclusion, *L. buchneri* DSM 32407 improved the reproductive performance of cows with **SCE** and healthy cows. This distinct lactobacillus strain seems to first have a stimulatory effect on the local immune system one week after its intrauterine administration to dairy cows on days 24–30 pp. This might be beneficial at that time point of the puerperium, presumably supporting the elimination of pathogenic bacteria from the uterine lumen. Three weeks after the administration, the endometrial mRNA expression of several pro-inflammatory factors was down-regulated, suggesting the containment of local inflammation possibly related to the earlier containment of pathogenic bacteria, which in turn might be associated with better parameters for reproductive performance. However, to confirm this suggestion of improved fertility, a larger cohort of cows treated with *L. buchneri* DSM 32407 would be necessary.

## Electronic supplementary material

Dataset 1
