# Supplementary material for: Influence of intrauterine administration of Lactobacillus buchneri on reproductive performance and pro-inflammatory endometrial mRNA expression of cows with subclinical endometritis
Source: Sci Rep. 2018 Apr 3;8:5473. doi: 10.1038/s41598-018-22856-y (PMC5883026; doi:10.1038/s41598-018-22856-y)
Supplement: Supplementary file 1 — Dataset 1 [file 41598_2018_22856_MOESM1_ESM.pdf]

# Influence of intrauterine administration of *Lactobacillus buchneri* on reproductive performance and pro-inflammatory endometrial mRNA expression of cows with subclinical endometritis

S Peter[1], M A Gärtner[1], G Michel[2], M Ibrahim[1], R Klopfleisch[3], A Lübke-Becker[4], M Jung[2], R Einspanier[1] and C Gabler[1,*]

[1]Institute of Veterinary Biochemistry, Freie Universität Berlin, Oertzenweg 19b, 14163 Berlin, Germany

[2]Institute for the Reproduction of Farm Animals, Bernauer Allee 10, 16321 Bernau, Germany

[3]Institute of Veterinary Pathology, Freie Universität Berlin, Robert-von-Ostertag-Straße 15, 14163 Berlin

[4]Institute of Microbiology and Epizootics, Freie Universität Berlin, Robert-von-Ostertag-Straße 7-13, 14163 Berlin, Germany

**Short title:** Influence of *Lactobacillus buchneri* on the bovine endometrium

**Key words:** *Lactobacillus buchneri*, endometritis, fertility, dairy cows

***Corresponding author:** Dr. Christoph Gabler

Institute of Veterinary Biochemistry

Freie Universität Berlin

Oertzenweg 19b

14163 Berlin, Germany

Tel.: +49-30-83862571

Email: christoph.gabler@fu-berlin.de

## Table and Figure legends:

**Supplement Table 1:** Selected gene transcripts, primer sequences and annealing temperatures used for real-time RT-PCR with resulting amplicon length.

**Supplement Table 2:** Number of samples positive after microbiological analysis of endometrial cytobrush samples from 31 cows of the **LAC** group and 17 cows of the **PLA** group at E1 (days 24-30 pp), E2 (days 31-37 pp) and E4 (days 45-51 pp).

## Supplement Table 1

| Gene | Primer sequence | Reference / GenBank accession no. | Fragment size | Annealing temperature |
|---|---|---|---|---|
| ***SDHA*** | For 5’-GGG AGG ACT TCA AGG AGA GG-3’ | [1] | 219 bp | 60 °C |
| | Rev 5’-CTC CTC AGT AGG AGC GGA TG-3’ | | | |
| ***SUZ12*** | For 5’-TTC GTT GGA CAG GAG AGA CC-3’ | [2] | 286 bp | 60 °C |
| | Rev 5’-GTG CAC CAA GGG CAA TGT AG-3’ | | | |
| ***CXCL1/2*** | For 5'-GAC CTT GCA GGG GAT TCA CCT C-3' | [1] | 125 bp | 60 °C |
| | Rev 5'-CGG GGT TGA GAC ACA CTT CCT G-3' | | | |
| ***CXCL3*** | For 5’-GCC ATT GCC TGC AAA CTT-3’ | [1] | 189 bp | 56 °C |
| | Rev 5’-TGC TGC CCT TGT TTA GCA-3’ | | | |
| ***CXCL5*** | For 5’-TGA GAC TGC TAT CCA GCC G-3’ | [3] | 193 bp | 61 °C |
| | Rev 5’-AGA TCA CTG ACC GTT TTG GG-3’ | | | |
| ***CXCR2*** | For 5'-AAC AGA CTC TGC CCC ATG TC-3' | [1] | 151 bp | 60 °C |
| | Rev 5'-AGT GAC AGA GCG ACC AAT CC-3' | | | |
| ***IL1A*** | For 5'-TCA TCC ACC AGG AAT GCA TC-3' | [4] | 300 bp | 59 °C |
| | Rev 5'-AGC CAT GCT TTT CCC AGA AG-3' | | | |

| | | | | |
|---|---|---|---|---|
| ***IL1B*** | For 5’-CAA GGA GAG GAA AGA GAC A-3’<br>Rev 5’-TGA GAA GTG CTG ATG TAC CA-3’ | [5] | 236 bp | 56 °C |
| ***IL6*** | For 5'-TCC AGA ACG AGT ATG AGG-3'<br>Rev 5'-CAT CCG AAT AGC TCT CAG-3' | [5] | 236 bp | 56 °C |
| ***IL8*** | For 5'-CGA TGC CAA TGC ATA AAA AC-3'<br>Rev 5'-CTT TTC CTT GGG GTT TAG GC-3' | [3] | 153 bp | 56 °C |
| ***IL10*** | For 5’-TGT TGA CCC AGT CTC TGC TG-3’<br>Rev 5’-TGG CTT TGT AGA CAC CCC TC-3’ | NM_174088 | 279 bp | 60 °C |
| ***IL1RN*** | For 5’-ATG TGG TAC CCA TCG AAC CC-3’<br>Rev 5’-AAC TTG GTG ACC TTG AGG GC-3’ | [4] | 298 bp | 59 °C |
| ***PTGS2*** | For 5'-CTC TTC CTC CTG TGC CTG AT-3'<br>Rev 5'-CTG AGT ATC TTT GAC TGT GGG AG-3' | [6] | 359 bp | 60 °C |
| ***PTGES*** | For 5’-TGC TGG TCA TCA AAA TGT ACG-3’<br>Rev 5’-GCA GTT TCC CCA GGT ATG C-3’ | [4] | 300 bp | 58 °C |
| ***PTGES3*** | For 5’-TGC AAA GTG GTA CGA TCG G-3’<br>Rev 5’-TAA CCT TGG CCA TGA CTG G-3’ | [4] | 253 bp | 61 °C |
| ***PTGDS*** | For 5’-TGA GAC GCG GAC CTT ACT G-3’ | [4] | 193 bp | 61 °C |

| | | | | |
|---|---|---|---|---|
| | Rev 5’-CTG GGA GCG GCT GTA GAG-3’ | | | |
| ***TNF*** | For 5’-CAA GTA ACA AGC CGG TAG CC-3’ | [7] | 354 bp | 60 °C |
| | Rev 5’-GCT GGA AGA CTC CTC CCT G -3’ | | | |
| ***MMP1*** | For 5’-GAT GAT GAT GAA TGG TGG ACC-3’ | [8] | 347 bp | 60 °C |
| | Rev 5’-TCC ACT TCT GGG TAC AAG GG-3’ | | | |
| ***PTPRC*** | For 5’-CAA ATT TAA ATG TGA TGG CGG-3’ | NM_001206523 | 286 bp | 56 °C |
| | Rev 5’-TCG TCC ACC TGG AGT AAT CC-3’ | | | |

## References


1 Gärtner, M. A. *et al.* Increased mRNA expression of selected pro-inflammatory factors in inflamed bovine endometrium in vivo as well as in endometrial epithelial cells exposed to Bacillus pumilus in vitro. *Reprod Fert Develop* **28**, 982-994 (2016).

2 Peter, S. *et al.* Puerperal influence of bovine uterine health status on the mRNA expression of pro-inflammatory factors. *J Physiol Pharmacol* **66**, 449-462 (2015).

3 Fischer, C. *et al.* Selected pro-inflammatory factor transcripts in bovine endometrial epithelial cells are regulated during the oestrous cycle and elevated in case of subclinical or clinical endometritis. *Reprod Fert Develop* **22**, 818-829 (2010).

4 Gabler, C. *et al.* Endometrial expression of selected transcripts involved in prostaglandin synthesis in cows with endometritis. *Theriogenology* **71**, 993-1004 (2009).

5 Konnai, S., Usui, T., Ohashi, K. & Onuma, M. The rapid quantitative analysis of bovine cytokine genes by real-time RT-PCR. *Vet Microbiol* **94**, 283-294 (2003).

6 Odau, S., Gabler, C., Holder, C. & Einspanier, R. Differential expression of cyclooxygenase 1 and cyclooxygenase 2 in the bovine oviduct. *J Endocrinol* **191**, 263-274 (2006).

7 Wijayagunawardane, M. P. B., Gabler, C., Killian, G. & Miyamoto, A. Tumor necrosis factor α in the bovine oviduct during the estrous cycle: messenger RNA expression and effect on secretion of prostaglandins, endothelin-1, and angiotensin II. *Biol Reprod* **69**, 1341-1346 (2003).

8 Bieser, B., Stojkovic, M., Wolf, E., Meyer, H. & Einspanier, R. Growth factors and components for extracellular proteolysis are differentially expressed during in vitro maturation of bovine cumulus-oocyte complexes. *Biol Reprod* **59**, 801-806 (1998).

**Supplement Table 2**

| | | **LAC** group (31 samples) | | **PLA** group (17 samples) | |
|---|---|---|---|---|---|
| | | **LAC/SCE** (16 samples) | **LAC/H** (15 samples) | **PLA/SCE** (15 samples) | **PLA/H** (2 samples) |
| | | Number of samples positive | | Number of samples positive | |
| **E1** | *Trueperella pyogenes* | - | - | - | - |
| | *Escherichia coli* | 2 | 1 | 4 | - |
| | *Histophilus somni* | - | - | 2 | - |
| | *Streptococcus uberis* | 1 | - | - | - |
| | *Klebsiella pneumoniae* | - | - | - | - |
| **E2** | *Trueperella pyogenes* | - | 1 | - | - |
| | *Escherichia coli* | 1 | 2 | - | - |
| | *Histophilus somni* | - | - | 3 | - |
| | *Streptococcus uberis* | 1 | - | 2 | - |
| | *Klebsiella pneumoniae* | 1 | - | - | - |
| **E4** | *Trueperella pyogenes* | 1 | 1 | 1 | - |
| | *Escherichia coli* | 1 | - | 3 | - |

| | | | | | |
|---|---|---|---|---|---|
| | *Histophilus somni* | 1 | 1 | 1 | - |
| | *Streptococcus uberis* | - | - | 1 | - |
| | *Klebsiella pneumoniae* | - | - | - | - |
